# Function and Psychotherapy of Chronic Suicidality in Borderline Personality Disorder: Using the Reinforcement Model of Suicidality

**DOI:** 10.3389/fpsyt.2020.00199

**Published:** 2020-03-18

**Authors:** Johannes M. Hennings

**Affiliations:** Department of Dialectical Behavioral Therapy, kbo-Isar-Amper-Klinikum Munich-East, Munich, Germany

**Keywords:** suicidality, borderline personality disorder, psychotherapy, reinforcement, behavioral analysis, suicide attempt, non-suicidal self-injury, DBT

## Abstract

Although great advancements in evidence-based therapies, chronic suicidal patients with borderline personality disorder (BPD) still challenge our mental health system. While BPD patients continue suffering from distress and aversive emotions, therapists and relatives feel often stunned and helpless when confronted with suicidality resulting in interruption of therapies, repeated presentations to emergency rooms and referrals to hospitals. Reviewing the current knowledge of the functions and background of non-suicidal self-injury, we learned that reinforcement mechanisms play an important role to understand why individuals act in deliberate self-mutilation. While individual motives for non-suicidal self-injury and suicidal behavior including suicidal ideations can differ, the principle mechanisms appear to be transferrable. Elucidating the individual motives and function of suicidal behavior is an important therapeutic step, giving us access to very central maladaptive schemes and false believes that we need to address in order to reduce chronic suicidality in BPD patients. This Perspective article aims to give a better idea of what is behind and what are the differences between non-suicidal self-injury, suicidal ideations and suicide attempts. It further integrates recent developments of behavioral science in a reinforcement model of suicidality that can provide therapists a practical armamentarium in their work with chronic suicidal clients.

## Introduction

Suicidality is one of the most alerting and urgent symptomatology in mental health. It summarizes a subset of psychopathological phenomena ranging from suicidal ideations (including thinking about, considering and planning suicide) to ambivalent suicide attempt, suicide attempt and suicide (1, 2). Across psychiatric disorders, risk factors for suicidal behavior such as childhood maltreatment, non-suicidal self-injury (NSSI), and previous suicide attempts have been identified (3, 4). Nevertheless, for various reasons [discussed in (5, 6)] the assessment of these factors (derived mostly from cross-sectional studies) does not sufficiently help predicting suicide risk in a patient at a given time (7). Further, suicide rates even increased in some populations such as adolescent girls during last years (5, 8). On the other hand, mental health care providers often refuse treating suicidal individuals or refer clients that become suicidal, with few evidence that treatment termination, referrals, or even involuntary hospitalization are effective in reducing suicide risk (6). Although we know, that the majority of suicide ideators will never act on their thoughts (9), in clinical practice, we do not have useful indicators (like fearlessness about death, subjective pain tolerance, and objective pain persistence) that help us to differentiate between suicide ideators and attempters (10). Further, NSSI often co-occurs in individuals with suicidal behavior, and although it is by definition not intended to be suicidal, clinical differentiation and appropriate managing can be demanding (3, 11). We further do not have good evidence for any pharmacological approach that addresses suicidality in these individuals (12). Consistently, patients with borderline personality disorder (BPD) that are among the individuals with the highest rates of chronic suicidal ideations (SI), NSSI and repeated suicide attempts, experience both, highly frequent hospitalizations and termination of treatment as a result of their high suicidality (13, 14).

Hence, what is behind chronic suicidality? What are the psychological mechanisms that maintain its chronicity? Can we apply concepts that helped us understanding and treating NSSI also for suicidal behavior? Can or should we do therapy in suicidal patients at all? And if yes, what are useful interventions?

### Suicidality and Non-Suicidal Self-Injury in Borderline Personality Disorder

NSSI (e.g., cutting, scratching, head banging, skin burning) is a world-wide phenomenon that occurs not only in BPD (15). Thanks to many studies and more precise definitions (i.e., “not intended to die”; discussed in [16, 17)], we acquired a much better understanding of the motives and background of NSSI during last years, even leading to a distinguished diagnostic entity in Diagnostic and Statistical Manual of Mental Disorders, 5th edition (18). As highlighted in the recent meta-analysis of Taylor et al. (19), we learned that NSSI can have a wide range of underlying functions within an individual (**Table 1**). We can distinguish intrapersonal functions like emotional regulation and self-punishment (escape/avoidance of internal states) from less prevalent interpersonal functions like interpersonal influence and peer bonding (3, 11, 19). Similarly, the different psychic functions of NSSI have been modeled by Nock & Prinstein (22) comparing positive (i.e., involves the addition of a favorable stimulus) versus negative (i.e., involves the removal of an aversive stimulus), and automatic (i.e., intrapersonal; e.g., emotion regulation) versus social (i.e., interpersonal; e.g., attention, avoidance-escape) reinforcing factors.

**Table 1:** Function of non-suicidal self-injury and suicide attempts.

Non-suicidal self-injury (3, 11, 19, 20)	Suicide attempt (20, 21)
Negative affect regulation, emotional regulation (most commonly reported [63–78% in (19)])	Emotional relief, relief of psychological pain [indicated from most patients in (20)]
Self-punishment	Interpersonal influence (may be less than at NSSI)
Anti-dissociation (e.g., causing pain to stop feeling numb)	To make others better off (much more than at NSSI)
Interpersonal influence (e.g., communicate distress; influence others behavior, actively hurt/punish others; less common [(33–56%) in (19)]	Sense of control
Anti-suicide (e.g., stopping suicidal thoughts)	
Sensation-seeking, distraction (e.g., doing something to generate excitement)	
Interpersonal boundaries (e.g., fitting in with others)	

Note that data derive from various means of assessment and item definitions across studies, ranging from structured and validated questionnaires to clinical interviews and case descriptions. For this reason, items that are likely to be related appear in one line. Data are compiled according to Brown et al., 2002, Klonsky (2007), Klonsky et al. (2013), the recent meta-analysis of Taylor et al. (2018), and Tullis (1998) (3, 11, 19–21).

These observations correlate nicely with recent neurobiological findings of NSSI, so that we can now retrace why patients repeatedly harm themselves - accepting necessity of surgical intervention, subsequent conflicts with their relatives, and even stigmatizing scars on their skin. In particular, NSSI reduces the activity of the amygdala while functional connectivity to the superior frontal gyrus is normalized in resting-state functional magnetic resonance imaging (23). Clinically, aversive tension decreases immediately after NSSI, and patients can think clearer again. It is further assumed that NSSI activates the reward system including the endogenous opioid system, presumably also the endocannabinoid system (24). In a prospective clinical study of frequently self-injuring BPD patients using continuous palmtop assessment of emotional states, Houben et al. (25) have impressively demonstrated the strong contingency between the occurrence of an aversive emotional state and subsequent NSSI. He further showed that beside the negative reinforcement of NSSI to relief emotional pain, NSSI reliably predicts the next aversive emotion (e.g., shame because having self-injured again/failed resolving the stressful situation), and subsequently the next and even further next NSSI during aversive emotional states, entering a vicious circle of repeated NSSI up to several times daily (25).

Clinically, we can use this background knowledge in therapy. For example, behavioral analysis like the stimulus-organismic-response-contingencies (SORKC or SORC) model of reinforcement-consequence (26) can illustrate to the patient in terms of psychoeducation that NSSI is a negative reinforcer (reduction of aversive tension) increasing the probability of NSSI in the next stressful situation. Most simply like in Dialectic Behavioral Therapy (DBT), the therapist can explain why it is necessary to stop NSSI and develop alternative means (“skills”) to reduce aversive tension in order interrupt the reward contingency received by NSSI (14).

### Can Suicidality in Borderline Be Conceptualized Similar to Non-Suicidal Self-Injury?

Brown, Comtois, and Linehan (20) were among the first investigating the background of NSSI in comparison to suicide attempts (SA). They found that in both cases, emotional regulation was a predominant function. In a between-person analysis, NSSI was more intended to generate feeling, self-punish, express anger and even distract, while SA was significantly more often intended to make others better off. Interestingly, self-punishment significantly differed between suicide attempters and non-suicidal self-injurers in the between-persons comparison, but not in the within-person comparison. The authors hypothesize that people who engage in non-suicidal acts intend to self-punish with both suicidal and non-suicidal parasuicide. Similarities and differences between NSSI and SA are depicted in **Table 1**.

Nevertheless, suicide attempts are just one symptom of suicidality and patients not conducting SA may still have frequent suicidal ideations or occupy with death and suicide in the internet and exchange suicide methods in social media (27). Indeed, SA typically occur in a circumscribed (early) phase during the course of BPD (28), whereas suicide ideations tend to persist over years (29). Fatally, it also turned out that highest suicide rates occur later in the course of the illness and follow long courses of unsuccessful treatment (30), meaning that patients are not at their highest risk of suicide when they are young and frequent visitors to the emergency room (29). Thus, working in therapy with chronic suicidality (beyond management of NSSI and SA) appears to be mandatory in order to prevent later suicides. At this point, the question rises whether we can simply adapt the reinforcement model described for NSSI to chronic suicidality? Can we assume similar contingency consequences for the appearance of suicide ideations in aversive emotional states? In other words:

### Can Thoughts Be Modeled Like Behavior in Psychotherapy?

**Figure 1:**
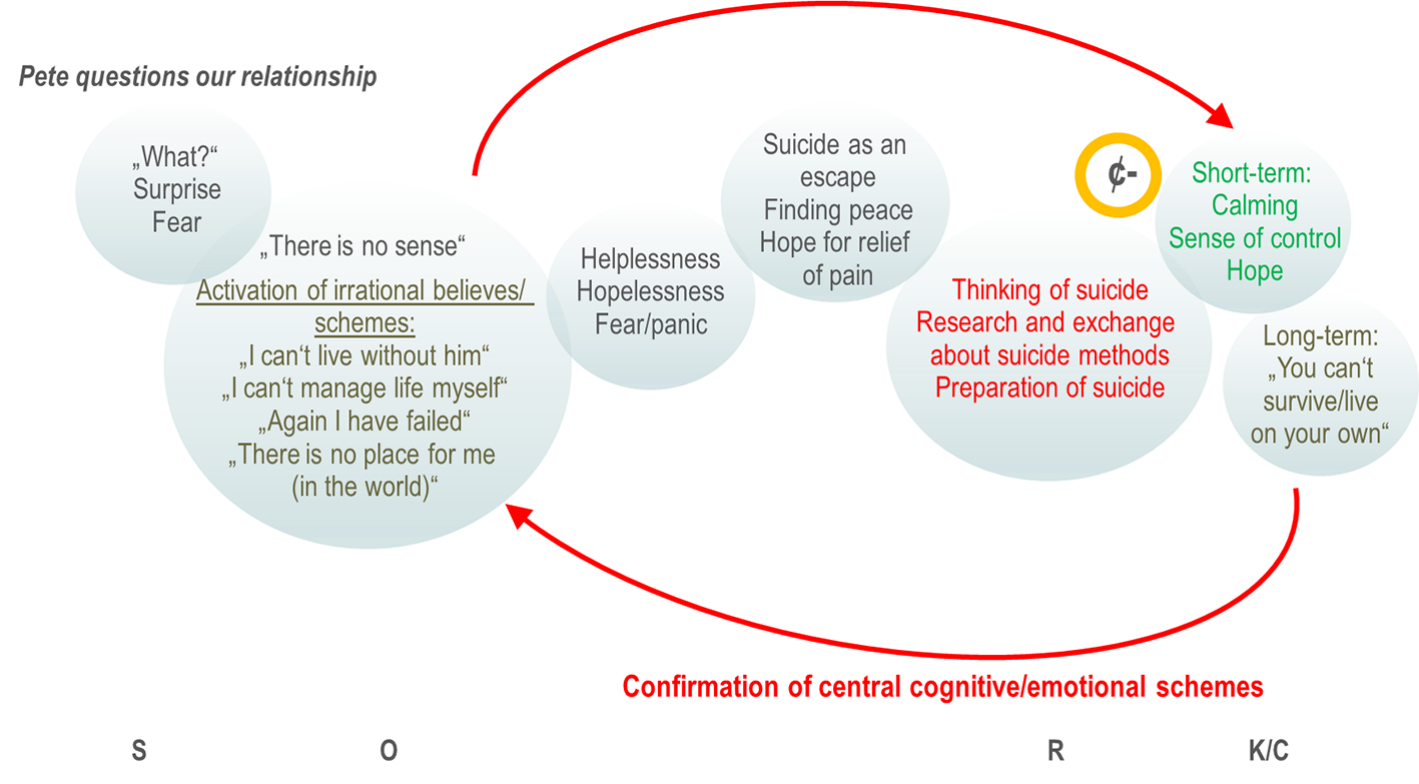
Chain analysis and stimulus-organismic-response-contingencies (SORKC) model of suicidal symptoms: A behavioral (chain) analysis of suicidal symptoms (e.g., suicidal ideation, internet research, preparation of suicide) that occur after the boyfriend of Jess questioned their relationship (cue, S). Jess instantly is surprised and fears losing Pete (primary emotions directly related to the situation; grief would be likely also). The first emotions disappear rapidly while Jess's maladaptive cognitive schemes (O) get activated (her interpretation of the situation against the background of her childhood experiences). These judgements in turn activate secondary emotions (like helplessness, hopelessness, panic) causing significant distress. Of note, these transitions from primary emotions to secondary emotions can be very fast letting patients even not notice their primary emotion at all (14). Thinking of suicide, the visit of suicide chats in the internet and suicidal communication with peers (R) calms Jess down and gives her a kind of feeling of control and hope (“I could escape,” “There is a way out,” “I must not suffer”). This contingency between psychological pain and relief (K/C) acts as a strong negative reinforcer (¢-) that increases the likelihood of suicidal ideations in the next situation of distress. On the other hand, long-term (i.e., after the immediate relief of pain) emotions like feeling of insufficiency, shame or loneliness occur (“I can't live alone,” “I am incapable in relationships,” “Anybody likes me,” “I am alone in the world”) that support in turn the assumptions/maladaptive schemes. The vicious cycle of reinforcement of suicidality and repeated confirmation of central cognitive/emotional schemes results in long-lasting, recurrent, chronic suicidality.

When we work with behavioral analysis in psychotherapy, we usually focus on (mostly) dysfunctional behavior, i.e., things that we have done, that have some kind of positive or negative consequences. As seen above, these consequences can reinforce me to act similarly the next time: Cutting in a stressful situation, e.g., will immediately reduce aversive tension (negative reinforcement) and may even give me the feeling of control over my emotions (positive reinforcement). But, is being absorbed to suicidal ideations really that different if these suicidal thoughts give me a kind of perspective, relief or just the idea that the current aversive situation will end? Chiles & Strosahl report that adolescents that experience intense emotional pain in response to internal (e.g., thinking of disabilities) or external stimuli can feel a kind of relief from the emotional distress when thinking about suicide (31). Similar to various behavioral patterns assigned to Hayes's so-called Experiential Avoidance (32), like NSSI, eating disorder or substance abuse that function to escape, avoid or modify an experience, suicidality can be regarded as a way to suppress emotions with suicide being the ultimate attempt at controlling psychological pain (33, 34). It was further Hayes (35), who integrated the obvious conceptual gap of internal processes (cognition) and behavior in his Relational Frame Theory (35) and, therapeutically, in the Acceptance and Commitment Therapy (ACT) (32). Assuming, according to the ACT theory, that human behaviors are functional, suicidality including thinking of suicide can be regarded as “a learned method of problem solving that involves escaping from or avoiding intense negative emotions” (31). Hayes states, that compared to classical reinforcement models (e.g., avoidance of closed rooms in agoraphobia, or relief from obsessional thoughts (e.g., contamination) by acting out compulsions (e.g., excessive hand washing), the relief in these situations is not directly conditioned (i.e., persons have not experienced that death releases emotional pain). Instead, individuals may have constructed “if … then” verbal associations [like “If I die, the bullying by my peers will stop” (36)]. This “verbal behavior”, enters a long-term conditioning processes, and may have aversive (negative) or appetitive (positive) consequences [see Murrell et al. for overview (33)]. Thus, applying this model to chronic suicidality in BPD, suicidal ideations (the verbal behavior) can reduce hopelessness, helplessness or unbearable anger and act as a negative reinforcer that will increase the probability of similar suicidal associations in an upcoming situation that produce similar aversive tension (**Figure 1**). The individual that experience a relief from suicidal ideations may feel an even bigger reinforcement value when he considers how, when, and where suicide would occur (31), letting him researching suicide methods in the internet, discuss suicide in online platforms, or even, when he prepares a suicide by collecting pills, looking for an appropriate place for hanging or a bridge to jump. The individual who ideates suicide, “from this perspective, experiences the ultimate reinforcement—a way to permanently and completely control difficult emotional experiences” (31).

### Contagious Suicidality?

It is frequently observed (for example in acute psychiatric settings) that BPD patients adopt dysfunctional behavior from other BPD patients (e.g., cutting although not having cutting before) indicating that the behavior must have a quite strong (at least short-term) positive consequence (they find out what helps best or they feel connected to peers that understand their need) (37, 38). One can speculate that exchange of suicide topics in online platforms is a similar phenomenon where patients try out different suicidal associations while discussing among each other. Not only can a suicidal association in Hayes's sense act as a negative reinforcer as described above (e.g., relief of pain), the exchange itself may become a kind of addictive behavior. In his theory of suicide addiction, Tullis (21) nicely describes these observations in his patients: Contemplating suicide can be pleasurable in some people, or, at least can be a break from psychological pain. Suicidal thoughts or behavior can be a form of self-medication in these individuals and reliving previous suicide attempts in thought or imaging death can become a ritual (or even trance-like) behavior providing a sense of control and an optional way out of pain. In his patients, Tullis further observed a calming sensation during suicidal thinking (up to a “rush,” “high,” “thrill,” “exhilaration”), they developed a tolerance to the effects of suicidal thoughts over time and engaged in compulsive rituals and behaviors, including secretly collecting and hoarding paraphernalia for suicide, characteristics we observe similarly in addictive disorders. Hence, these behavioral observations support the hypothesis that in some individuals, occupation with suicide is pleasurable, reduces pain and becomes “a way of life” (39) - psychologically spoken, are object to reinforcement and contingency loops.

### Judgments and Beliefs Are the Toxic Ingredients of the Reinforcement Model of Suicidality

When we look out for a new approach addressing suicidality in BPD, then working with the background and motives becomes vital in the proper sense. In the language of behavioral analysis, it is the organism variable that determines how we rate and react upon upcoming stimuli (40, 41). Besides biological factors, the organism variable is largely influenced by experiences we made, messages we became when we were a child or parent models we had (42). Similar to our clinical example in **Figure 1**, suicidal adolescents and BPD frequently share believes of being worthless, inadequate, rejected or blameworthy resulting from invalidation or traumatization (33, 42). Typical examples in these cases are: “I can't live alone,” “I am false,” “I don't have a place in the world,” “I am bad,” “I can't handle it.” They are robust convictions of themselves and the outside world or automatic thoughts (43) that become activated (in their conscious mind) through internal or external cues (e.g., when they are or feel offended, disappointed, rejected, lonely,…). As shown recently, dependent of the severity of BPD, aversive emotional states can then highly contingently linked to a specific dysfunctional behavior (e.g., intense anger after being offended, or NSSI after being disappointed) (44).

Using behavioral analysis, the therapist may elucidate reinforcing contingencies within the vicious chain of cues, activated faulty believes and subsequent suicidal ideations. He further can look for more adaptive behavioral alternatives or identify possible obstacles that inhibit the application of functional behavior. Typical obstacles can be, e.g., intensive emotions of fear, shame or guilt, or faulty believes and assumptions (e.g., “I am a looser,” “I have no right”) (14). The latter is probably the most important aspect that helps understanding the background and function of suicidality in these patients.

Similar to the reinforcer model of NSSI described above, we can speculate that the confirmation of central schemes together with the reinforcement of suicidal ideations stabilizes the dysfunctional system of chronic suicidality. Conversely, in NSSI on the other hand, the rapid dynamic of the next aversive emotional states directly after NSSI exemplified in the Houben study (25) acts as a punishment in the behavioral sense, resulting presumably in an earlier fade out of self-injuring behavior after some time (as frequently observed in the courses of BPD), while chronic suicidality persists.

### Interventions Deduced From the Reinforcement Model of Suicidality

Addressing the motives and psychic function behind suicidal behavior depending on specific situations (or triggers) may be a first, but potentially very powerful step in the therapeutic work with suicidal BPD patients. They may feel a substantial validation by going through their individual behavioral analysis and by understanding their own reinforcement mechanisms (**Figure 1**). As described by Murrell (33), normalization of suicidal ideations or behavior with respect to the individual's situation (“If I were in this situation, I would think/feel similar.”) and in comparison to others (“Many people at your age have had serious thoughts about killing themselves – it isn't that uncommon or weird.”) can reduce shame (about not getting along with the challenges of life, e.g.) and helps establishing acceptability of discussing suicide in an honest and genuine way. Working with faulty believes and assumptions may be one of the most challenging, but on the long run, inevitable approaches in psychotherapy of chronic suicidality. Cognitive techniques as well as emotional exposure in order to reach a cognitive and emotional reappraisal may be applied here (14, 42). As proposed in ACT, so-called defusion techniques (i.e., distancing and disconnecting techniques from thoughts and feelings) can be a highly relevant addressing negative judgements and believes, too (33). Given the high prevalence of traumatization and substantial invalidation experiences in chronic suicidal individuals, exposure-based trauma therapy has an important impact on suicidal symptoms and, according to recent developments in DBT for posttraumatic stress disorder can (and most likely should) start as soon as possible (45, 46). Within this confrontation, time for the grieving process and finally acceptance of what has happened in the past is inevitable. For this process, ACT and the compassion-focused therapy provide useful assistance (32, 47). In parallel, it becomes important to establish alternative non-suicidal behavior that at least at the beginning is reinforced with help from the therapist. On the long run, the goal is to establish a naturally reinforcing system, e.g., by using values of the patient: building up and connection to a circle of friends, feeling of conjointness by taking responsibility and social integration (i.e., volunteering, taking care of a pet,…). According to Hayes, also “verbal behavior” can enter such long-term conditioning processes, like “If I live, my parents might get to see me graduate from college someday,” or “If I kill myself, it would really hurt my family to go to my funeral” [taken from (33)]. Without claiming completeness, the **Supplemental Table** gives an overview of possible interventions that can be derived from the behavioral analysis of reinforcement (**Supplemental Table 1**). These interventions comprise standard behavioral techniques as described in DBT, ACT, and compassion-focused therapy, including validation techniques, psychoeducation, cognitive techniques, emotional regulation, and the development of alternative behavior and skills. The proposed approaches are mostly adapted to the clinical example depicted in **Figure 1**, but they may be transferred also to other BPD patients with chronic suicidality.

## Discussion

We have seen that similar to NSSI, suicidal ideations and behavior can have various motives and functions. Overall, these functions appear to have in common to significantly reduce mental pain, be it by reducing aversive tension, by giving an idea of a way out of the current situation, or by giving a sense of control of difficult emotions (like guilt, shame, intense anger). Thus, it appears plausible that reinforcement mechanism as presented here are substantially involved not only in NSSI, but also in chronic suicidality. Intriguingly, a recent functional MRI study strongly substantiates this concept from a neurobiological perspective using autobiographic transcripts to recall patients' previous suicidal episodes: In this paradigm, mental pain triggering suicidal behavior is associated with decreased prefrontal activity whereas planning and acting out suicidal impulses (in mind) in response to mental pain is associated with increased activity in the medial prefrontal cortex, the anterior cingulate cortex, and the hippocampus suggesting that goal-directed suicidal behavior is associated with a reduction of mental pain (48).

Nevertheless, there is still a big gap in the literature delineating the continuum from passive suicidal thoughts, ideations of dying or being death to suicide attempt preparation and definite suicidal acts, and the psychological function of each of these suicidal behaviors may be very different within and between individual subjects. Indeed, there is strong evidence from neurobiology, that NSSI, SI, and SA have very distinct (and sustained) effects on the regulation of the hypothalamic-pituitary-adrenal (HPA) hormonal axis, a system that is essentially important in adaptation to challenging situations in life. It has been shown, for example, that cortisol response in the combined dexamethasone suppression/corticotropin releasing hormone stimulation test is attenuated in both, past and recent suicide attempters compared to suicide ideators or non-suicidal patients in major depression (49). In another most recent study, the interaction of a HPA axis response with psychosocial stress differentially predicts suicidal behavior and ideations within 18 months, with, again, a lower cortisol response being associated with suicidal behavior (50). Interestingly in this regard, epigenetic mechanisms have been claimed to be involved in the neurobiology of suicidality including the HPA axis regulation (51) which may possibly explain some sustained effects observed in recurrent suicide attempters. The meaning of these findings and its implementation to psychological models of suicidality definitively needs further investigation. The challenge of future research will thus be to combine sophisticated methods form both, neurobiology and psychology. In both cases, clear differentiation and definitions of suicidal symptoms is of eminent importance.

From the clinical perspective too, a thorough assessment of all degrees of suicidality becomes crucial for the therapist when estimating the individual patient's risk to proceed from ideating to acting in suicide during treatment. In particular, he wants to know, which factors pushes the patient form ideation to suicidal action. Fearlessness about death and pain tolerance occurs in several suicide models as a factor differentiating suicide ideators from attempters (3, 4, 9, 52, 53), and most robust predictors of SA identified in studies may be closely related to these items as they reflect a previous experience of loss of physical integrity (e.g., NSSI, history of previous SA, childhood maltreatment). They can indeed be helpful for the therapist estimating the patient's individual risk of progression from ideation to attempts, with connectedness being one of the most important protective factor in this respect (52). Nevertheless, the reliability of the prediction (i.e., the negative predictive value in this case) may be too low to exclude a suicide risk, and the results of recent studies have questioned the ability of such factors to robustly distinguish suicide ideators from attempters across diagnostic entities including student samples (10, 54–57). Further, what to do in psychotherapy with BPD patients that are carriers of suicide risk factors? The assessment of predictors alone does not give an answer to this question. But, without doubt, these patients in particular should be subject to a psychotherapy addressing their suicidality. There is a common understanding in third wave behavioral therapies [discussed in (42)], that new behavior or alternative experience can only be learned or made by the brain when it is done in the same or a similar situation that normally would have cued the old (dysfunctional) behavior. In other words, the patient needs to act (or think) differently in a situation he normally becomes suicidal. And, at this point, referral to a hospital would likewise not allow a new experience (but will confirm old assumptions: “I have failed again,” “I can't handle it on my own,” “I am punished because I behave badly,” “suicide is the best option”). Instead, the therapist needs to assist him just then: In the real situation (e.g., during a crisis), or in activated states of critical emotions (of whom the therapist knows its link to the patient's suicidal behavior) in a therapy session, the therapist needs to guide the patient to regulate its emotions, reflect the situation and help applying new behavior. For this purpose, the therapist may offer telephone or online coaching, techniques of emotional regulation and stress tolerance, as well as thorough behavioral analysis of suicidal behavior and thinking. Nevertheless, this kind of work with suicidal patients is demanding and indispensably needs a secure frame also for the therapist. Apart from a common commitment of both sides (“going the new way,” “finding a way to stay alive,” “being ready for exposure”) the use of non-suicide contracts, individual crisis plans and agreements about contingencies (what happens after NSSI, SA, therapy-interfering behavior,…) have been strongly recommended for the psychotherapy of chronic suicidality in BPD (14).

Although we have learned a lot about suicidality during last years and specific programs from different therapeutic schools including behavioral and psychodynamic approaches (58) helped many individuals, the implementation of validated anti-suicidal interventions and suicide prevention for a larger number of affected people is still needed. Educative suicide prevention programs using the internet and social media have now been launched addressing the need of low-threshold communication with individuals at risk and further aiming to increase suicide-prevention-related knowledge (59, 60). Nevertheless, understanding the individual background of suicidality takes time, a trustful therapeutic relation and non-judgmental attitude of the therapist. The proposed reinforcement model of suicidality applies basic behavioral techniques to chronic suicidal BPD patients. It is not a new model. It integrates theoretic concepts that helped us understanding related phenomena like NSSI and suicide attempts. It further includes known interventions that have been efficient in suicidality and the treatment of BPD, such as DBT and ACT. But, it especially stresses the role of reinforcement of suicidal ideations and behavior, thus giving us tools to work with the patient and to find a shared commitment for planned interventions in order to dissolve suicidal contingencies. Albeit, it is of vital importance to further investigate the background of suicidality, especially with respect to all forms of suicidal ideations and behavior in chronic suicidal patients. Precise definitions and assessments appear to be crucial in these studies. Further, many of the here mentioned techniques have not specifically been tested upon its ability to modify central dysfunctional schemes or reinforcements so far. Modern neurobiological techniques like fMRI, neuroendocrinology or epigenetics in combination with appropriate psychological paradigms may help us to further prove our concepts, detect new options for anti-suicidal interventions and hypothetically specifically monitor therapeutic effects.

## Author Contributions

The author confirms being the sole contributor of this work and has approved it for publication.

## Conflict of Interest

The author declares that the research was conducted in the absence of any commercial or financial relationships that could be construed as a potential conflict of interest.

## Abbreviations

ACT, acceptance commitment therapy; DBT, dialectical behavioral therapy; HPA, hypothalamic-pituitary-adrenal axis; NSSI, non-suicidal self-injury.
