# Supplementary material for: Function and Psychotherapy of Chronic Suicidality in Borderline Personality Disorder: Using the Reinforcement Model of Suicidality
Source: Front Psychiatry. 2020 Mar 18;11:199. doi: 10.3389/fpsyt.2020.00199 (PMC7093558; doi:10.3389/fpsyt.2020.00199)
Supplement: Supplementary file 1 [file Table_1.pdf]

Supplemental Table 1: Possible therapeutic intervention that can be deduced from a behavioral analysis of suicidality. Of note, the proposed interventions are closely related to/taken from interventions proposed in DBT and ACT for suicidal behavior (1,2) as well as compassion-focused therapy (3). Nevertheless, the proposed scheme can be applied also for suicide ideations and other suicidal behavior beyond suicide attempts (e.g., research of methods, preparation, chat). As already stated by Linehan (1993), an analysis of how the patient actually avoids suicidal behavior can be extremely useful defining opportunities for the therapist to reinforce alternative problem-solving behaviors (1).

	**Stimulus (S)**	**Organism (O)**	**Response (R), consequence contingency (K/C)**
	I.e. antecedents, cues, internal/external triggers (problematic events)	*I.e. faulty believes, assumptions, biological factors that made me vulnerable in the situation (temperament, current depression, sleep deprivation, drugs, childhood maltreatment...)*	*I.e. dysfunctional behavior/thoughts, reinforcement mechanisms*
Validation techniques	Validation of primary emotions (i.e. emotions that come first and are suitable to the situation/stimulus)	Validation of individual vulnerability<br>Instruction of self-validation techniques (e.g. „This is really a difficult situation for me“, “This was a shock”)	Validation of emotions that preceded the suicide ideations (e.g. distress, pain, helplessness)<br><br>Validation of reinforcement (“I understand that thinking of suicide gave you a little bit of hope in this moment”)
Psychoeducation	Identify and name primary emotions (often, they are not present to the patient as secondary emotions come that rapidly)	Psychoeducation (e.g. which personal experiences make me vulnerable to suicidal ideations/behavior? What are my faulty believes?)	Explaining the function of suicidal behavior in terms of pain relief or regulation of aversive emotions (“reward”, reinforcement)<br><br>Explaining the vicious cycle and the effect that suicidal ideations/behavior confirm and maintain central maladaptive cognitions/schemes.
Cognitive techniques	Discrimination learning techniques (e.g. “what is now different to the difficult situation in childhood when I felt such helplessness, too”)	Mindfulness techniques (e.g. awareness of automatic thoughts, secondary emotions, physiological reactions (e.g. heartbeat, weakness, numbness…)<br><br>Cognitive reappraisal of faulty believes (“Although my intensive feelings let me think that I can’t stand it any longer, I know that I have	Pro-/con-lists about suicidal ideations/behavior and alternative thoughts/behavior<br><br>Constructing thoughts/images of positive futures (e.g. “The New Way” in DBT including values, needs and desires)<br><br>Commitment strategies as proposed in ACT or

		survived similar situations in the past already"; "I am o.k.",…)<br><br>Defusion techniques (i.e. distancing and disconnecting techniques from thoughts and feelings)	DBT (e.g., giving the life a chance (for a certain time at least), giving the commitment of staying alive for even a short period of time (e.g. until the next appointment next week)
Emotional regulation	Emotional exposure of primary emotions/cue situations (providing nonreinforced exposure: reattribution of S-R contingency)	Emotional regulation of secondary emotions (emotions that are evocated by internal judgments/interpretations: e.g., helplessness or shame after an unexpected situation that causes fear)<br><br>Exposure-based trauma therapy like DBT-PTSD (4,5)	Blocking avoidance behavior (disrupting reinforcement contingencies)
Alternative behavior, skills	Block avoidance behavior: e.g. staying in the situation, adequate expression of feelings and needs ("This makes me really sad", "I don't want to lose you", "Can you help me understanding your decision?")	Alternative techniques for self-reassurance and self-pacification	Therapist reinforces alternative problem-solving behavior, non-suicidal responses, maintains position that suicide is not a good solution, generates hope.<br><br>Over time attention should be faded to ensure that resistance to suicidal behavior comes under the control of natural reinforcers (1):<br><br>Built of alternative functional behavior that is (naturally) reinforced by attractive rewards (e.g. build up and connection to a circle of friends, feeling of conjointness by taking responsibility and social integration (i.e. volunteering, taking care of a pet…)).

References

1. Linehan MM. *Cognitive-Behavioral Treatment of Borderline Personality Disorder*. Guilford Publications, New York, NY (1993).

2. Hayes SC. "Verbal relations, time, and suicide," in *Understanding verbal relations*, eds. S. C. Hayes, L. J. Hayes. Context Press, Reno, NV (1992) 109–118.

3. Gilbert P. *Compassion focused therapy: Distinctive features*. Routledge, New York, NY (2010).

4. Bohus M, Schmahl C, Fydrich T, Steil R, Müller-Engelmann M, Herzog J, Ludäscher P, Kleindienst N, Priebe K. A research programme to evaluate DBT-PTSD, a modular treatment approach for Complex PTSD after childhood abuse. *Borderline Personal Disord Emot Dysregulation* (2019) **6**:7. doi:10.1186/s40479-019-0099-y

5. Bohus M, Dyer AS, Priebe K, Krüger A, Kleindienst N, Schmahl C, Niedtfeld I, Steil R. Dialectical behaviour therapy for post-traumatic stress disorder after childhood sexual abuse in patients with and without borderline personality disorder: a randomised controlled trial. *Psychother Psychosom* (2013) **82**:221–233. doi:10.1159/000348451
